# Experiences of Multiple Myeloma Patients With Treatment in the Palestinian Practice: A Multicenter Qualitative Study in a Resource-Limited Healthcare System

**DOI:** 10.7759/cureus.63365

**Published:** 2024-06-28

**Authors:** Thabet Zidan, Hala Iskafi, Ahmad Ali, Husam Barham, Mahdi Al-Sayed Ahmad, Raed Masalma, Ahmed Hossoon, Ali Barham, Ramzi Shawahna

**Affiliations:** 1 Faculty of Medicine and Health Sciences, An-Najah National University, Nablus, PSE; 2 Department of Physiology, Pharmacology and Toxicology, Faculty of Medicine and Health Sciences, An-Najah National University, Nablus, PSE; 3 Clinical Research, An-Najah National University Hospital, Nablus, PSE

**Keywords:** resource-limited country, healthcare systems, qualitative research, infection control, communication, multiple myeloma

## Abstract

Background

Multiple myeloma is a crippling cancer that puts a significant strain on patients and their families alike. The long and exhausting treatment journey with the disease is challenging not only for patients but also for healthcare systems. This exploratory study was conducted to look into these patients’ experiences with their treatment and explore their recommendations and views to improve the Palestinian healthcare system, which can be viewed as an evolving healthcare system within a resource-limited and developing country.

Methods

The consolidated criteria for reporting qualitative research (COREQ) checklist was used for conducting this multicenter exploratory qualitative study. A total number of eight patients with multiple myeloma who received treatment in the Palestinian healthcare system participated in semi-structured in-depth interviews. The semi-structured in-depth interviews followed a set interview schedule. Thematic analysis of the data was done using the qualitative interpretive description approach.

Results

A total of 5.48 h (329 min) of total interview time was analyzed. Among the patients, 6 (75%) were males, 5 (63.5%) lived in urban areas, 5 (62.5%) reported satisfaction with their household income, 6 (75%) underwent bone marrow transplantation, and all of them (100%) had governmental insurance. The qualitative data that emerged after analysis were classified into three major themes and multiple sub-themes. The three major themes were: (1) treatment side effects, (2) factors affecting treatment experience, and (3) recommendations to improve healthcare service.

Conclusion

The results of this qualitative study offer insight into how people with multiple myeloma view the healthcare system in Palestine and shed light on the variable and challenging experiences with their treatment, side effects, and communication with healthcare providers within the context of a resource-limited and developing country. Future research should involve hemato-oncology doctors and benefit from their expertise in the field.

## Introduction

Multiple myeloma is a proliferative clonal plasma cell cancer that is characterized by abnormally increased monoclonal immunoglobulins [1]. Despite all of the success for most multiple myeloma patients, 25% of newly diagnosed patients live for less than 3 years [2]. Myelosuppression, peripheral neuropathy, infections, fatigue, and gastrointestinal disorders are the most frequent side effects brought on by multiple myeloma treatment [3]. Secondary cancers, infections, and reductions in quality of life are some of the long-term negative effects of allo-hematopoietic stem cell transplantation (allo-HSCT) [4]. Contrarily, autologous HSCT (auto-HSCT) is widely used to strike a balance between the risks and benefits because it is safer than allo-HSCT and more successful than conventional chemotherapy; despite the fact that it has a high recurrence rate that cannot be disregarded [5]. In addition to chemotherapy, prophylaxis and supportive treatment are essential parts of the therapeutic management of multiple myeloma patients [6].

Healthcare is provided in the Palestinian territories by four major providers: the Ministry of Health (MoH), the United Nations Relief and Works Agency for Palestine Refugees (UNRWA), non-governmental organizations (NGOs), and the private sector [7]. The Palestinian Authority pays for the full costs of cancer treatment, as patients get treated in either governmental or non-governmental institutions, or through a referral to Israel or any other country [8]. Referrals are calculated as a part of the operational expenditure section, which constitutes a significant percentage of the total health expenditure as reported by the Palestinian MoH, with oncology referrals making up almost 25% of the total specialties needed for referrals [9, 10]. The ongoing political situation makes the fragmentation and supply shortages in the Palestinian healthcare system worse [11]. In addition, Israeli restrictions on obtaining the necessary permits for the Palestinian population exacerbate the problem, and oncology patients suffer disproportionately [9,11-13]. Only 61% of Gaza patients had their medical permits issued in 2021 compared to 88% of West Bank patients [9].

There is a lack of studies that address multiple myeloma in Palestine or any other resource-limited and developing countries, and particularly there is no sufficient data that pertains to the nature of the treatment these patients receive and the different factors that affect treatment experience. In this study, we focused on these issues and investigated the various experiences of multiple myeloma patients as well as the standard of treatment they got within the framework of the Palestinian healthcare system as an example of a limited-resources healthcare system. Moreover, the advantages of this research could also apply to people with other hematologic malignancies.

## Materials and methods

Study design

This exploratory study was conducted and reported using the consolidated criteria for reporting qualitative (COREQ) checklist, which is a set of unified criteria for reporting qualitative research [14]. Semi-structured in-depth interviews with multiple myeloma patients receiving treatment in the Palestinian healthcare system were conducted. A predetermined interview schedule was followed for the semi-structured in-depth interviews. Half of the patients were interviewed at their houses, and the other half were interviewed in private rooms at the hospital.

Recruitment of interviewees

The study participants were located, invited, and recruited with the help of hemato-oncologists who provide care for this subset of patients. The approach of purposive sampling was applied. This method was used to ensure that the patients in this study meet the inclusion criteria due to the rarity of multiple myeloma and the specialized nature of the target group. The patients were chosen from the main referral center where multiple myeloma patients receive their treatment in Palestine. The participants were invited based on the following inclusion criteria: (1) patients who had been diagnosed for more than 1 year to ensure they had sufficient time to highlight their experience with their disease, (2) being more than 18 years old, (3) willing to participate in audio-recorded interviews, and (4) providing written informed consent. Patients who had diseases that affected their cognitive function and those who were not willing to participate in audio-recorded interviews were excluded.

Number of interviews needed for this study

Thematic saturation was used to establish the interview’s endpoint. The sample size required for this investigation was predetermined because this method is adaptable. According to earlier research using thematic saturation as an interview endpoint [15-17], at least 5 h of interview time would be required to achieve conceptual thematic saturation with major themes, sub-themes, and patterns. Thus, with a median of 40 min per interview, we would need at least eight interviews to saturate the thematic data.

Collection of qualitative data

A preliminary search of the scientific literature reporting on the treatment of multiple myeloma was conducted before the interview schedule was developed. The researchers developed an interview schedule based on earlier research on multiple myeloma [18-20]. The interview schedule gathered demographic data from the patients, such as gender, age, the amount of time since their multiple myeloma diagnosis, where they lived, how satisfied they were with their household’s income, and how satisfied they were with their social life. Additionally, the interview schedule featured open-ended questions and prompts that allowed the interviewers to push the subjects to provide more details and the participants to speak for a considerable amount of time. Four researchers and hemato-oncologists assessed the interview schedule to verify face and content validity. The final interview schedule kept the questions that the four panelists determined to be acceptable. Discussions and agreement were used to settle differences in the panelists’ perspectives. After getting training from the research supervisor (RS) on how to conduct semi-structured in-depth interviews, three final-year medical students from An-Najah National University (TZ, AA, and HI) interviewed the patients, as TZ and AA were male, and HI were female.

Before conducting each interview, the researchers affirmed that the study was conducted as scientific research and that none of them had any desire to affect the study’s conclusions. Both the interviews and the field notes were recorded. The substance of the interviews was verbatim recorded. Each participant gave their written, informed consent. Since every interview was audio-recorded, there was no need to repeat any of them.

Data analysis

The researchers read over the transcripts multiple times to have a general comprehension of their content. The interview content was examined qualitatively using the interpretative description technique [21,22]. The interpretive description methodology is known to enable the finding of important themes, sub-themes, and patterns when compared to other qualitative analytic methodologies, especially when dealing with complicated topics like those found in healthcare. Key themes, sub-themes, and patterns were additionally identified using the Leuven Qualitative Analysis Guide [23]. The ability to organize the qualitative data into major themes, sub-themes, and patterns was made possible by associations and similarities among the data points. Bracketing was employed to keep the analysis credible and trustworthy. To avoid any preconception bias, the researchers made every effort to keep their own knowledge, experiences, and expectations to themselves during the investigation [24]. The researchers kept and scrutinized audit records of the analytical decisions to ensure reliability. To ensure accurate categorization of the error situations into the main categories and subcategories, the researchers triangulated the qualitative data from each interview script through multiple readings of the transcripts, routine discussion among the researchers, consensus to resolve contentious decisions, and repeated readings of the transcripts [25].

Ethical considerations

The Institutional Review Board (IRB) of AnNajah National University approved the study protocol in its entirety as well as its ethical values. In addition, consent was obtained from the hospital’s management, ethics, and/or research committees. The conduct of the study adhered to the Declaration of Helsinki’s standards for scientific and medical research. Each participant in this study gave written informed consent before participating.

## Results

Interviews were conducted with eight multiple myeloma patients. The total interview time was 5.48 h (329 min), and the median interview duration was 43.5 min.

General characteristics of the patients

Among the patients, six (75.0%) were males, five (62.5%) were city dwellers, four (50.0%) were 60 years or older, five (62.5%) reported satisfaction with household income, and six (75.0%) underwent bone marrow transplantation. The detailed characteristics of the interviewees are shown in Table 1.

**Table 1:** The general characteristics of the interviewees (n = 8)

Characteristic	n	%
Gender		
Male	6	75
Female	2	25
Age (years)		
< 60	4	50
≥ 60	4	50
Underwent bone marrow transplantation		
Yes	6	75
No	2	25
Time elapsed since diagnosis (months)		
< 24	4	50
≥ 24	4	50
Place of residence		
Urban	5	62.5
Rural	3	37.5
Self-rated satisfaction with household income		
Unsatisfied	3	37.5
Satisfied	5	62.5
Self-rated satisfaction with social life		
Unsatisfied	3	37.5
Satisfied	5	62.5

Results of the content analysis

When the scripts were analyzed, three major themes and multiple sub-themes emerged. The major themes were: (1) treatment side effects, (2) factors affecting treatment experience, and (3) recommendations to improve healthcare service.

Treatment Side Effects

Patients reported various side effects to treatment, ranging from mild to life-threatening side effects. The most commonly reported side effects were diarrhea, infections, fatigue, loss of appetite, hair loss, kidney dysfunction, and anemia. These side effects had a major impact on patients’ physical and mental well-being.

When asked about their expectations regarding the adverse outcomes of the proposed treatments, most patients reported that prior to undergoing treatment they had the opportunity to have a thorough conversation that dissected the important questions about the treatment such as the benefits and expected adverse outcomes. These conversations were of utmost importance for the patients as they enabled them to formulate a comprehensive understanding of what was going on which eased their minds, alleviated their doubts, and made them feel prepared to manage expected as well as unexpected side effects. Nevertheless, despite the provided communication, in some instances, some patients reported feeling frustrated about being informed inadequately about potential side effects (Table 2).

**Table 2:** In-depth patient quotes on reported side effects, discussion of side effects with healthcare providers, and benefits of communicating with healthcare providers

Topic		Patient quote
Reported side effects	Diarrhea	“I had severe diarrhea for 9 days after they started giving me the high-dose chemotherapy to destroy the bone marrow. The diarrhea was very severe. Anything I would eat or drink would be out within minutes… It was so bad that I put a chair at the bathroom’s door! I would be going back and forth from the chair to the bathroom.... I lost so much weight during that time. I remember begging the doctors and telling them: (For God’s sake! It is all diarrhea, please stop it!)...” (Male, age 58, diagnosed in 2019)
	Infections	“I had many viral infections. Once, I had herpes zoster, and it was followed by neuralgia. The pain remained for three months. These three months were full of pain and agony. I didn’t leave any drug that I didn’t try, even the antidepressants… I also had proctitis, one of the drugs I was taking is known to cause it… These infections were the real deal. If you tell me about things like vomiting, nausea, and I don’t know what else, these things are really soft compared to the infections I experienced. All of these side effects happened to me, and I forgot about them. Thank God that one can forget these things…” (Male, age 35, diagnosed in 2020)
	Fatigue	“The worst side effects for me were fatigue and fever. They were so hard that I thought I would never survive them!” (Male, age 62, diagnosed in 2020)
	Loss of appetite and nausea	“I had a loss of appetite and nausea. Whenever they cooked something in the house and I smelled it, I felt like I wanted to throw up… They would bring the food to me and I just couldn’t eat. My sisters thought that it was up to me not to eat, but in fact I just couldn’t…” (Female, age 58, diagnosed in 2021)
	Hair loss	“During the treatment, I lost all my hair, even my beard. That was very difficult to the point that I started having nightmares about the hair loss... This is when I truly understood what chemotherapy meant. Also, because everyone, even without having any medical background, can see that I am bald and I probably have the bad disease (cancer)…” (Male, age 35, diagnosed in 2020)
	Kidney dysfunction	“The thing that affected me the most was when my creatinine increased. I was fatigued and could not stand. It felt like my whole body was collapsing…” (Male, age 60, diagnosed in 2020)
	Anemia	“The worst thing I experienced was the reduction in blood level after chemotherapy. I started feeling dizzy and lost my balance. This affected me psychologically because I didn’t understand why this was happening. I had severe anxiety and I couldn’t sleep because I kept thinking about why this was happening to me. They gave me sleeping pills to help me sleep because I went 3 days without sleeping a single minute…” (Male, age 67, diagnosed in 2020)
Discussion of side effects with healthcare providers		“I spoke with my doctor. We used to follow up every two weeks with my doctor in my hometown. I used to inform him of any side effects I faced, and he would clarify them and explain why I was experiencing them. The nurses also informed me of any potential side effects of the medications before they would administer them.” (Male, age 60, diagnosed in 2020)
		“I asked the doctor about my vision deterioration, and he told me that it is ok, and that it’s not caused by chemotherapy… They didn’t explain it might become worse, they didn’t explain these side effects to me…” (Male, age 58, diagnosed in 2019)
Benefits of communicating with healthcare providers		“I benefited from the communication with doctors and nurses. I believe it made me aware of all the potential outcomes and what I should expect after every treatment.” (Male, age 55, diagnosed in 2021)
		“Yes of course I benefited from it [communicating with healthcare providers]. One relieves the doubt he has when he knows what he is experiencing…” (Male, age 62, diagnosed in 2020)
		“I felt at ease after talking with the doctors. I was able to prepare for the side effects and protect my body from them, like drinking water to prevent kidney damage. I felt better having known these side effects might occur before experiencing them.” (Male, age 60, diagnosed in 2020)

Factors Affecting Treatment Experience 

Most patients expressed trust in undergoing treatment in the Palestinian healthcare system. They respected and valued the efforts made by healthcare providers despite the substantial workload and pressure they were under. However, one patient had a negative experience with her bone marrow transplantation and was advised against undergoing this procedure in Palestinian hospitals. Moreover, patients appreciated the fact that they were not burdened with the costs of their cancer treatment, which has greatly alleviated their financial stress. Patients also reported that the referral system operated efficiently, with referrals being processed quickly and smoothly and no delays reported. However, concerns were raised over the political situation and the security restrictions, which posed some challenges for one patient causing a delay in his treatment.

Patients expressed concerns regarding infection control within healthcare facilities. They observed many instances where cleaning workers used the same equipment to clean both patients’ rooms and bathrooms. In addition, it was observed that cleaning workers lacked proper education and training on infection control guidelines, which compromised the safety of some patients. Moreover, one patient mentioned being surprised to find another patient in the same room during transplantation, raising concerns regarding the hygienic practices of fellow patients. Furthermore, it was reported that waiting rooms were overcrowded and many patients did not wear facial masks; thereby increasing infection risk and causing discomfort among patients (Table 3).

**Table 3:** In-depth patient quotes on factors affecting treatment experience

Topic	Patient quote	
Exploring trust	“The healthcare service is very good and ensures comfort and respect for the patient. So, I feel comfortable getting treatment here. The healthcare workers take care of the patients even though they are under a lot of pressure.” (Male, age 62, diagnosed in 2020)	
	“If not for my trust in it (the healthcare system), I would’ve looked for treatment outside of this country, through a referral to Israel, or without a referral to any other country.” (Male, age 35, diagnosed in 2020)	
	“I don’t advise anyone to do bone marrow transplantation in this country... It is not easy to collect the cells (stem cells) from patients! (The patient started crying here) After all the suffering I went through, my doctor told me that my operation was unsuccessful… It was like you picked me up and threw me to the ground!...” (Female, age 58, diagnosed in 2021)	
Insurance and coverage	“The only thing I respect in this country is the healthcare system, because they really do their job to the best level. Whoever enters the hospital doesn’t pay a penny, and gets the whole treatment whether they are rich or poor, old or young, it is the same thing. This is the best feature of the Palestinian healthcare system…” (Male, age 62, diagnosed in 2020)	
	“Since I was diagnosed with the disease, I have not faced any financial difficulties… They (the government) took care of the disease and its treatment completely…” (Female, age 58, diagnosed in 2021)	
Referrals and delays	“There was no trouble at all (getting a referral)! At first, I thought it was very quick because I am a government employee, but in fact, it is the same for all cancer patients. The process was very quick in an impressive way, and I did not enter the routine lengthy referral process. I had no trouble at all!” (Male, age 35, diagnosed in 2020)	
	“There were no delays, quite the opposite in fact, they (referral office workers) were the ones in a hurry.” (Male, age 62, diagnosed in 2020)	
Political situation	“They told me they have to transfer me to another hospital in Israel to get an accurate diagnosis, but I told them I can’t go to any hospital in Israel or even Jordan, because of security restrictions owing to my political views… They told me to figure it out by myself! I went to the Palestinian liaison (the official body responsible for security coordination with Israel through its District Coordination Offices (DCO)) to get a referral on my own, and it took almost 3 weeks to go through!… Once, I had an appointment at Al-Makassed Hospital (based in Jerusalem), but my permit had expired. So, I took the risk and went in the bus (which transfers cancer patients from Nablus city to Jerusalem hospitals). Luckily, the soldiers only looked at my photo and didn’t notice the dates!” (Male, age 58, diagnosed in 2019)	
		Infection control	“A cleaning worker enters the bathroom and he cleans it. Then, with the same wiper, he enters my room and starts cleaning it! He destroyed everything!” (Male, age 35, diagnosed in 2020)		
“When the cleaning workers enter the room, they would get close to me with their spoiled equipment. I used to tell them to put their masks on, but they usually giggled or laughed! These workers are usually not well-educated, and they don’t understand these things…” (Male, age 58, diagnosed in 2019)			
“What annoyed me was that they told me I would be alone in the room (during the bone marrow transplantation), but I was surprised when I found another patient in the room! We are supposed to clean and disinfect everything when we use the bathroom, but how would I know if the other patient did that?” (Male, age 58, diagnosed in 2019)			
“One hospital’s waiting area is too small for the number of patients. The patients would be sneezing and coughing, and some of them weren’t wearing face masks…” (Female, age 58, diagnosed in 2021)			

Recommendations to Improve Healthcare Services 

Based on the opinions and experiences of the patients, a number of recommendations were made to enhance the healthcare services. The need for increased infection control training, particularly for cleaning staff, was stressed by patients. They emphasized how crucial it is to engage other individuals who deal closely with patients in the training programs. Patients also recommended the establishment of NGOs to help cancer patients who are struggling financially, especially those who have dependents to support.

Patients reported limitations with the electronic medical records system in the Palestinian hospitals, including regular errors and lags that caused delays and issues for patients. Patients underlined the need for system upgrades to increase the system’s effectiveness. Patients also recommended hiring more healthcare professionals, particularly nurses, to lessen their workload and provide higher-quality care. In addition, patients demanded that healthcare services be provided in one place to avoid having to travel between various facilities and cities for various treatments. They believed that combining healthcare services in one location would make it more comfortable for patients, especially those who had comorbidities. Finally, patients suggested individualizing meal requirements during inpatient stays, particularly for patients with comorbidities like diabetes or kidney failure (Table 4).

**Table 4:** In-depth patient quotes on their recommendations to improve healthcare services

Topic	Patient quote
More training on infection control	“The existing protocols are directed at healthcare workers. A lot of training courses for doctors, a lot of training courses for nurses… But other workers? It is not only doctors and nurses who work with me, the cleaning worker goes in and out of the room too… They (cleaning workers) should receive training as well!” (Male, age 35, diagnosed in 2020)
Financial support for cancer patients	“Look, not everyone has the same financial abilities. It is true that patients don’t pay (for treatment), but for their families and others it can be costly, especially when the provider stops bringing in money… If there would be some societal organizations that take care of these people… There are some people with tough tumors, and they have families to provide for. So, there must be some societal organizations to help these people until the provider gets back to work…” (Male, age 62, diagnosed in 2020)
Improving the electronic medical records system	“…Also, the computer keeps lagging. There would be a lot of patients and the computer is lagging. This causes more trouble… When you have 150 patients waiting for their turn in the clinic, but they are delayed because the computer keeps lagging… They should do something about it, it is very annoying!” (Female, age 58, diagnosed in 2021)
Increasing number of healthcare workers	“I feel there is an error in the management and administration of hospitals. One time I needed to have a cannula, I waited for the nurse for almost an hour since she had too many patients to look after… If they can add more nurses this would really help the patients.” (Male, age 62, diagnosed in 2020)
	“I believe the patients with multiple myeloma should receive special care… The hospital management should increase the number of nurses so their workload decreases…” (Male, age 55, diagnosed in 2021)
Defragmentation of healthcare services	“At the start of every month, I have to go to the primary healthcare center to get my medications, but two of them (medications) are in a hospital and the other medication is in another healthcare center that is far from the first hospital! Why don’t they make all the medications available in the main hospital where I do routine blood tests every month? This would save a lot of time and effort!” (Male, age 67, diagnosed in 2020)
Individualizing diet needs during inpatient stays	“The hospital staff should be more considerate of the patients’ different conditions like diabetes and kidney failure. The food shouldn't be the same for all patients. I have diabetes, I can’t eat everything they serve to all other patients…” (Male, age 58, diagnosed in 2019)

## Discussion

Understanding how cancer patients perceive their symptoms and engage with the healthcare system is essential for healthcare providers and decision-makers. It allows them to design appropriate interventions to improve the quality of life and experiences of these patients. In this particular study, the focus was on patients with multiple myeloma and their experiences within the Palestinian healthcare system, which serves as an example of healthcare systems in developing and resource-limited countries. The study specifically explored how these patients perceived the side effects they experienced, communicated their concerns, and recommended improvements. This qualitative study is the first of its kind to investigate the experiences of multiple myeloma patients in the Palestinian healthcare system or any similar region.

Patients reported different side effects with chemotherapy and bone marrow transplantation alike, and these side effects include but are not limited to constipation, diarrhea, hair loss, fatigue, loss of appetite, numbness, and infections. Similar side effects have been reported in relevant studies [18-20], and patients emphasized the profound difficulties they faced trying to cope with these side effects. Patients communicated their concerns over these side effects with healthcare workers. The majority felt more prepared when they knew the side effects before they occurred, and some felt more at ease after speaking with healthcare workers about them. It is of great importance that patients understand the risks and benefits of treatment [26]. It was observed that patients who report having better communication experiences have been found to have better health outcomes; hence, improving patient communication is essential to raising the quality of care and its outcomes [27]. Effective communication with healthcare providers might be a crucial tactic to aid cancer survivors in coping with the effects and unpredictability of the disease [28]. Furthermore, it has been emphasized that cancer patients find it of paramount importance to communicate with healthcare workers from the moment of recurrence until the end of treatment [29]. Clinicians should encourage the discussion about treatment side effects and advise rest, exercise, and sleep, as well as address the possible treatments that might be helpful [30].

Most patients had trust in being treated in the Palestinian healthcare system and by Palestinian healthcare professionals, and they expressed that healthcare workers do their best job despite all the pressure they are put under. It was reported in the literature that higher trust in healthcare professionals leads to better health behaviors, fewer symptoms, higher quality of life, and more treatment satisfaction [31]. Increasing patient trust in healthcare can be done through emphasizing quality, communication, and information [32]. For some patients, insurance and coverage were the best features of the Palestinian healthcare system, as oncology patients are completely covered financially by the government. Financial burdens are common among cancer patients and are associated with worse treatment adherence and quality of life [33].

Most patients had no delays and saw the referral process as simple and quick, while a patient reported he had problems and delays because of his political views. Delay in cancer treatment is a problem in the health system worldwide. It was reported that even a 4-week delay in treatment has been associated with increased mortality rates; thus, policies focused on reducing delays to treatment initiation could improve survival outcomes [34]. The ongoing political situation in Palestine affects the health of the Palestinian population, as it was reported that Israeli authorities’ denial of referrals has led to unnecessary deaths and suffering, especially for cancer patients [12,13].

Patients reported different concerns and recommendations in the healthcare system. Many patients had problems with infection control, emphasizing problems with crowded waiting rooms and mistakes made by housekeeping staff, as one of the patients recommended more training for them. Infections are a major leading cause of death in multiple myeloma patients [35], and it was shown that training of cleaning workers can help decrease nosocomial infection rate [36,37].

Patients also recommended hiring more healthcare workers to help lessen the workload on doctors and nurses, as Palestine has a ratio of 14.9 general physicians, 6.7 specialized physicians, and 27.9 nurses per 10,000 population [38], and it was shown that increased numbers of healthcare workers lead to improved care and decreased mortality [39,40]. Other recommendations included offering financial assistance to cancer patients because multiple myeloma patients face significant financial challenges in the areas of employment, disability, and retirement [41]. As a result, healthcare professionals must be aware of their patients’ financial situations in order to quickly intervene to lessen the effects of multiple myeloma and its treatment. Improvement of the medical records system that has a problem with lagging was also recommended, along with assessing each patient’s individual nutritional requirements based on their condition and comorbidities; which is crucial in the treatment process and will lead to better survival, quality of life, and lower complications rate [42]. Patients also recommended receiving healthcare in one place instead of fragmentation of care, which is linked to higher mortality, longer lengths of stay, and higher readmission risk [43]. Multiple myeloma puts significant stress on patients and their families, significantly affects quality of life, and poses a challenge to healthcare systems that function in resource-limited and developing countries. It is vital to address these patients’ experiences with their treatment in the realm of the Palestinian healthcare system, as the healthcare system is known to suffer from insufficient resources, fragmentation, and inadequate medical supplies [7].

Strengths and limitations

This exploratory investigation provided many advantages. As a result of a thorough analysis of the literature, the interview schedule first covered practically every topic related to the study’s goals. Second, respondents were questioned in complete privacy at their homes or in private hospital rooms. A family member was typically there to assist the patient in recalling certain aspects or to participate in the conversation. Third, the patients were diversified based on age, gender, time since diagnosis, place of residence, and income level. Thus, this diversity should have added more strength and rigor to our qualitative data. Fourth, this is the first qualitative study conducted in Palestine that focuses on patients with hematologic malignancies in general and multiple myeloma patients in particular. The study’s findings can assist healthcare professionals and policymakers in endorsing initiatives that will improve the quality of care for these patients.

The limitations of our study should be taken into account when interpreting or applying the findings. Although the small sample size (n = 8) is acceptable for a qualitative study, the findings cannot be applied to the entire population of multiple myeloma patients. Additionally, this study used a qualitative approach, which, while effective in describing patients’ experiences with the healthcare system, is inappropriate for quantifying certain aspects like side effects or recurrences. Instead, a quantitative approach should have been used to describe these aspects. Additionally, healthcare professionals were not included in this study, which would have produced more data and added to it.

Although bias cannot be completely eliminated, the rigor, credibility, and authenticity of the data gathered for this study should have been improved by the use of audio recordings of the interviews, bracketing, audit trail reviews, multiple readings of the transcripts, frequent discussion among the researchers, using consensus to settle contentious decisions, and triangulation of the qualitative data.

## Conclusions

The study emphasizes how multiple myeloma patients are significantly impacted by side effects of cancer treatments, such as fatigue, infections, and diarrhea. Communication with healthcare providers helped many patients with their expectations, but some felt misinformed. Patients valued the efforts of healthcare professionals and the financial support from medical authorities, and they viewed the Palestinian healthcare system with general trust. Although delays were occasionally caused by the complicated geopolitical situation in Palestine, effective referrals were also observed. There were serious worries about infection control and crowded waiting rooms. Patients suggested that in order to enhance their treatment experience, hiring more healthcare personnel, de-fragmentation of healthcare services, individualized meal plans, improved infection control training, financial assistance for patients in need, and changes to the medical records electronic system should be implemented. Future studies should include healthcare professionals and appropriately consider their perspectives on the subjects covered in this study.
